# Towards the elimination of dog-mediated rabies: development and application of an evidence-based management tool

**DOI:** 10.1186/s12879-020-05457-x

**Published:** 2020-10-20

**Authors:** Kristyna Rysava, Tamara Mancero, Eduardo Caldas, Mary Freire de Carvalho, André P. B. Castro, Veronica Gutiérrez, Daniel T. Haydon, Paul C. D. Johnson, Rebecca Mancy, Lúcia R. Montebello, Silene M. Rocha, Jesús F. Gonzalez Roldan, Marco Antonio Natal Vigilato, Victor Del Rio Vilas, Katie Hampson

**Affiliations:** 1 University of Warwick, School of Life Sciences, Gibbet Hill Road, Coventry, UK; 2 University of Glasgow, Institute of Biodiversity, Animal Health and Comparative Medicine, Graham Kerr building, MVLS, Glasgow, G12 8QQ UK; 3 Pan American Health Organization (PAHO), Duque de Caxias, Rio de Janeiro, Brazil; 4 Virology, Central Laboratory, State Center for Health Surveillance, State Department of Health, São Paulo, Rio Grande do Sul Brazil; 5 Ministry of Health, Brasília, Distrito Federal Brazil; 6 Ministry of Health, Mexico, Distrito Federal Mexico; 7 University of Surrey, School of Veterinary Medicine, VSM Building, Guildford, UK

**Keywords:** Canine rabies, Decision support tool, Freedom from disease, Interruption of transmission, Management recommendations, Mass dog vaccination, Scientific guidance, Surveillance

## Abstract

**Background:**

International organizations advocate for the elimination of dog-mediated rabies, but there is only limited guidance on interpreting surveillance data for managing elimination programmes. With the regional programme in Latin America approaching elimination of dog-mediated rabies, we aimed to develop a tool to evaluate the programme’s performance and generate locally-tailored rabies control programme management guidance to overcome remaining obstacles.

**Methods:**

We developed and validated a robust algorithm to classify progress towards rabies elimination within sub-national administrative units, which we applied to surveillance data from Brazil and Mexico. The method combines criteria that are easy to understand, including logistic regression analysis of case detection time series, assessment of rabies virus variants, and of incursion risk. Subjecting the algorithm to robustness testing, we further employed simulated data sub-sampled at differing levels of case detection to assess the algorithm’s performance and sensitivity to surveillance quality.

**Results:**

Our tool demonstrated clear epidemiological transitions in Mexico and Brazil: most states progressed rapidly towards elimination, but a few regressed due to incursions and control lapses. In 2015, dog-mediated rabies continued to circulate in the poorest states, with foci remaining in only 1 of 32 states in Mexico, and 2 of 27 in Brazil, posing incursion risks to the wider region. The classification tool was robust in determining epidemiological status irrespective of most levels of surveillance quality. In endemic settings, surveillance would need to detect less than 2.5% of all circulating cases to result in misclassification, whereas in settings where incursions become the main source of cases the threshold detection level for correct classification should not be less than 5%.

**Conclusion:**

Our tool provides guidance on how to progress effectively towards elimination targets and tailor strategies to local epidemiological situations, while revealing insights into rabies dynamics. Post-campaign assessments of dog vaccination coverage in endemic states, and enhanced surveillance to verify and maintain freedom in states threatened by incursions were identified as priorities to catalyze progress towards elimination. Our finding suggests genomic surveillance should become increasingly valuable during the endgame for discriminating circulating variants and pinpointing sources of incursions.

## Summary

International agencies have committed to the goal of global elimination of dog-mediated rabies. Considerable progress towards eliminating dog-mediated rabies has been achieved in Latin America; however, the region has encountered setbacks and further work is still needed to achieve this goal. Scientific guidance for managing rabies control programmes to ensure that setbacks are overcome and that progress continues during the endgame is critical, yet often limited and lacking in specific recommendations. Characteristic patterns of disease dynamics are indicative of progress towards elimination, and through their identification, tailored guidance can be provided. Here, we develop a robust tool to evaluate progress from routinely collected surveillance data and to inform rabies elimination programmes of where and how surveillance and control efforts need improvement. We developed the tool using the surveillance database for rabies in Latin America (SIRVERA) maintained by the Pan American Health Organization. We demonstrate the utility of this tool to support policymakers and rabies programme managers at regional, national and subnational levels through its application in Mexico and Brazil. We further developed an interactive web interface for communicating this progress and guidance (https://boydorr.shinyapps.io/paho_rabies/), which can be applied throughout Latin America and other regions around the world as regional rabies elimination programmes mature using their established surveillance systems. Priorities highlighted by applying this tool in Mexico and Brazil were to: strengthen the delivery and monitoring of dog vaccination campaigns in identified persistent foci; and to enhance surveillance to distinguish virus variants, to support rapid response to incursions and to verify disease freedom.

### Motivation

Rabies has been eliminated from domestic dog populations in high-income countries, but remains a major public health concern in low- and middle-income countries. Every year, thousands of people die and billions of dollars are lost due to rabies spread by domestic dogs [1]. Regional and national targets for the elimination of dog-mediated rabies have now been set [2] and control programmes are underway around the world [3–5]. A suite of resources is available to support countries in the global campaign to eliminate human deaths from dog mediated-rabies by 2030 [6], spanning the entire process from zoonotic disease prioritization [7, 8], calculating programmatic resource needs [9] to validation of zero human deaths and verification of interruption of transmission and rabies freedom [10, 11]. Many of these tools are accessible from the Rabies Blueprint Platform (www.rabiesblueprint.org), a live document hosting up-to-date and comprehensive case studies, procedures and protocols for rabies control and prevention [12]. These complementary tools can be used strategically to foster sustainable collaborations for rabies elimination [13]. In particular, the Stepwise Approach towards Rabies Elimination (SARE) is for use by countries to self-assess their national programmes and should be repeated periodically to benchmark progress and revise priorities [14]. However, for countries or regions with already well-established rabies control programmes including surveillance systems generating data on programme impacts, there is limited guidance on how to interpret those data and tailor activities accordingly to ensure progress towards elimination remains on track.

### Rabies management in Latin America

Most progress has been made towards the regional elimination of dog-mediated rabies in Latin America. Since 1983, national dog vaccination programmes coordinated by the Pan American Health Organisation (PAHO) have controlled canine rabies across much of the Western Hemisphere, reducing incidence by over 99% [3]. But, as the region approaches elimination, differences in progress have emerged [15]. Variability in the implementation of control measures, as well as geographic, population and socioeconomic differences likely underpin differential progress in rabies control. However, observed patterns may also reflect variation in the quality of surveillance. Clarifying the relationship between these influences and their effects on rabies detection and circulation is key to designing effective interventions during the endgame. For example, some areas have ostensibly achieved rabies freedom and now face competing priorities that create pressure to reduce expenditure on rabies control. In contrast, other areas are struggling to control rabies, and continued circulation poses a risk for reintroduction into neighbouring areas, potentially threatening the success of the regional programme [16, 17]. Consequently, there is an increasingly urgent need to address these differences within the region and for targeted scientific guidance to ensure continued progress. By tailoring efforts to local epidemiological situations, it should be possible to accelerate progress towards elimination and sustainable freedom from disease.

### Management tool

We present a tool for programme managers and practitioners working at regional, national and subnational levels to guide their programme management using routine surveillance data from their local area and from neighbouring areas that may influence their epidemiological situation. The tool was designed using data from SIRVERA, a regional rabies surveillance database first established in 1969 (http://sirvera.panaftosa.org.br/). As such, the tool is targeted towards countries or regions with already well-established rabies control programmes and surveillance systems with regular submission of samples from suspect animals. Our principles in developing this tool were that: (1) it should be possible to classify the epidemiological situation objectively in defined geographic areas using routine surveillance data; (2) categories and their criteria should be easy to understand; (3) classification should provide insights into the effectiveness of current management and guidance for further progression, including readiness to undertake independent verification of rabies freedom [10, 11]. Here we describe the rationale for the tool, its methodological development and assessment of its robustness. We apply this tool sub-nationally across Mexico and Brazil, where dog rabies control programmes have been ongoing since the 1980s. Using the countries’ routine surveillance data, we reveal historical and current patterns of rabies circulation, and generate guidance for surveillance and control strategies that are tailored to specific localities to facilitate progress towards elimination.

## Methods

### Algorithm development

Our aim was to develop a tool to help rabies managers and practitioners to understand progress towards rabies elimination by distinguishing areas with ongoing transmission from areas where efforts have controlled rabies and have potentially interrupted transmission. To be both useful for programme managers and epidemiologically meaningful, we envisaged a tool for application across relatively large administrative units such as states, provinces or districts, rather than smaller units such as villages or municipalities. We focused on case reports (i.e. numbers of laboratory confirmed cases per unit area and per unit time), as this is the simplest information recorded in most surveillance systems for rabies. In addition, we also identified scenarios where viral characterization, specifically identification of the viral variant of the detected case, provides additional clarity. We developed a classification algorithm to evaluate and distinguish characteristic patterns when applied to extended periods (minimum of 5 years) of complete monthly surveillance data. In the process of refining the classification algorithm through application to states (major sub-national administrative units) in Brazil and Mexico, we discussed the resultant classifications with state-level and national stakeholders to clarify our interpretation.

### Data

For development of the algorithm we used data on laboratory confirmed rabies cases in dogs from Mexico and Brazil submitted to SIRVERA between January 2005 and December 2015. The raw data are publicly available from the SIRVERA website or can be shared on request from sirvera@paho.org. Both countries have ongoing rabies control programmes that were initiated in the 1980s, with annual dog vaccination campaigns managed at the state-level, and major declines in laboratory confirmed rabies cases recorded since the start of their programmes [18]. These countries were selected by PAHO due to the quality of their surveillance, which was considered adequate for this classification both at national and state level. This qualitative assessment was based on previously recorded declines in incidence, continued detection of wildlife rabies variants and submission of samples, lack of reported human rabies deaths from canine rabies, diagnostic laboratory proficiency and completed monthly records in SIRVERA. SIRVERA has recently been updated to capture information on virus variants. However, since this information had not been routinely recorded in SIRVERA previously, data on virus variants were provided separately by the Ministries of Health in Mexico and Brazil.

### Classification criteria

**Fig. 1:**
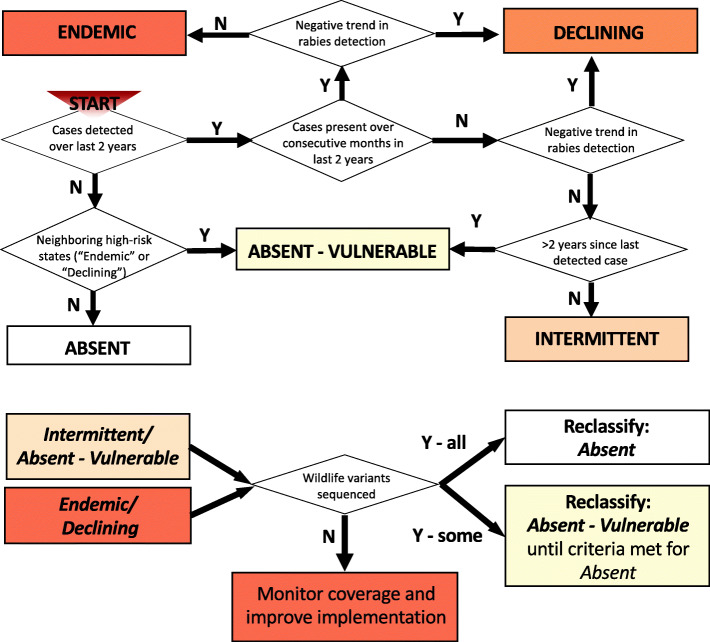
Classification algorithm. For use in settings with established dog rabies control programmes including routine annual mass dog vaccination and adequate surveillance. Algorithm includes reclassification step based on variant assessment

We developed the algorithm to classify second-level administrative units (hereafter referred to as states) into 5 putative categories: *Endemic*, *Declining*, *Intermittent*, *Absent-Vulnerable* and *Absent* (Table 1), based on characteristic patterns identified via a set of objective criteria (Fig. 1, Table 2). We caution that classification to the categories *Absent-Vulnerable* and *Absent* does not satisfy OIE recommendations for declaration of freedom. The classification criteria and their rationale are outlined below.

**Table 1:** Putative epidemiological classifications

**1) ENDEMIC TRANSMISSION:** Canine rabies (variants 1 & 2 in Latin America [19]) detected over at least two consecutive months during the previous 2 years, indicating focal transmission. No significant decrease in the frequency of months with case detection over the previous 5 years. **2) DECLINING TRANSMISSION:** At least 1 month with detected canine rabies cases in the previous 2 years, but a declining frequency of months with detected cases over the previous 5 years. **3) INTERMITTENT DETECTION:** Canine rabies cases detected during the past 2 years but not over consecutive months. No temporal trend in the frequency of months with detected cases during the previous 5 years. **4) ABSENT-VULNERABLE:** Either: (i) canine rabies cases not detected in the previous 2 years, but neighbouring an area where rabies is *Endemic* or *Declining* and therefore vulnerable to incursions; or (ii) a single month with cases detected during the previous 2 years, but no case detection prior to that month for at least 2 years (i.e. recently experienced an incursion that did not lead to further spread). **5) ABSENT:** No cases of canine rabies cases detected during the last 2 years and minimal risk of incursion (i.e. not neighbouring with any *Endemic* or *Declining* states).

**Table 2:** Classification criteria (corresponding to Fig. 1). NA - not applicable, V1 and V2 - canine rabies genetic variants of type 1 and 2 respectively in Latin America [19]

Classification	Cases in last 2 years	Trend (model coefficient) over 5 years	Absence (> 2 yrs with no V1 or V2)	Incursion risk
**Endemic**	Yes, V1 &/ V2, over at least two consecutive months	None/ positive	NA	NA
**Declining**	Yes, V1 &/ V2	Negative	NA	NA
**Intermittent**	Yes, but not over two or more consecutive months	None/ positive	NA	NA
**Absent - Vulnerable (i) or (ii)**	(i) No (ii) V1 &/V2 in 1 month only	(i & ii) NA	(i) At least last 2 years (ii) > 2 yr absence prior to last detected case(s)	(i & ii) Adjacent to *Endemic* or *Declining* area(s)
**Absent**	No	NA	NA	Not adjacent to any *Endemic* or *Declining* areas

#### Case detection

For each state, we calculated the time since the last detected case, and categorized case detection in the last 2 years as: present in at least two consecutive calendar months; present, but not over consecutive months; and absent (Criterion I). This criterion was used together with trends in case detection (Criterion II) to distinguish between *Endemic*, *Declining* and *Intermittent* classifications. States where rabies was detected in a single month during the last two years but that had an absence of cases prior to that month for at least 2 years were classified as putatively *Absent-Vulnerable*. Among states with no case detection in the previous 2 years, we distinguished between two categories (*Absent* and *Absent-Vulnerable*) according to incursion risk (Criterion IV).

The rationale for Criterion I was based on the following arguments. Firstly, following an introduction, the serial interval for rabies means that ~35% of secondary cases are expected to occur within 1 month. A lack of detection over consecutive months therefore suggests secondary transmission is not sustained and no other introductions occurred. A lack of detection could also result from inadequate surveillance, but in combination with temporal trends in case detection (Criterion II), this would result in an *Intermittent* classification. Our decision to use a two-year window for the classification of *Absent* is based upon modelling work showing that while mass dog vaccinations are ongoing, a two-year period without detection of rabies, should be sufficient to be confident of elimination, even at realistically low levels of surveillance [20].

#### Temporal trends in case detection

We used monthly state-level time series of laboratory confirmed cases spanning several years of surveillance data to assess temporal trends. After converting these time series to monthly state-level binary data (presence-absence), we fitted a logistic regression to determine whether the monthly probability of case detection was increasing, decreasing or showed no trend (Supplementary Figures S1). Using binary data in this way (as opposed to case counts) aims to overcome spurious inference from either fluctuation in incidence or detection that may reflect awareness, investment or other external drivers (see section on Algorithm Testing and Validation, and Supplementary Figures S1 and S2). Since cases are detected during the month when rabid animals become infectious, but infection could also be present prior to this due to latent infections we adjusted the time series for our regressions to include the month prior to case detection as presence. If cases were detected over at least two consecutive calendar months (excluding the latent period) in the past 2 years, we classified states with time series exhibiting significant declines in case detection as *Declining*, versus those with no trend or an increasing frequency of months with detected cases as *Endemic*. For states where cases were detected in the past 2 years but not over consecutive months (excluding the latent period), we classified the states as either *Declining* or *Intermittent* depending upon whether the data exhibited decreasing or no temporal trend in detection. It should be noted that for the classification of *Declining* to apply, we required that a state be implementing annual (or more frequent) dog vaccination campaigns.

#### Variant identification

Cases detected in the previous 2 years were assessed to determine whether they were due to variants associated with dogs (V1 & V2) or with other species (e.g. bats, terrestrial wildlife). Classifications were then updated with wildlife-associated variants removed. A reclassification to *Absent* or *Absent-Vulnerable* could therefore occur if all detected cases from the last 2 years were due to wildlife variants. This criterion provides a check as to whether a sylvatic virus may have spilled over into domestic dogs and whether further investigation is required to understand complexities in transmission and maintenance.

#### Incursion risk

We assessed the risk of incursions into non-endemic *Absent* states based on shared borders with high-risk states or countries (*Endemic or Declining*). We classified as *Absent-Vulnerable* those states with at least one neighbouring state classified as either *Endemic* or *Declining* (Table 2).

### Algorithm testing and validation

We subjected our classification algorithm to robustness testing and validation. Initially we applied our algorithm to SIRVERA data from 2005 to 2015 for Mexico and Brazil, classifying states in both countries retrospectively using a rolling multi-year time window. To explore how the length of the time series affected the sensitivity of the logistic regression approach to identify trends, we refitted to incrementally truncated rolling windows of presence-absence data (from 2 to 10 years), and compared classifications according to how many years of data were included. For temporal trends, we explored the possibility of modelling incidence (case counts) using Poisson regression rather than Logistic regression (presence-absence timeseries) (Figure S1). We also considered whether missing information (major and minor administrative units corresponding to states and municipalities respectively) would indicate inadequate surveillance and the impact of missing information on classifications in Mexico and Brazil.

To assess how well our algorithm performed under different levels of surveillance and/or reporting quality, we simulated canine rabies dynamics, and then resampled our simulated time series to mimic differing levels of surveillance before applying our classification algorithm.

To simulate canine rabies, we adapted an existing model [21]. Our aim was to simulate epidemiological dynamics not specific to a particular setting, but for populations of a similar size and scale to those in states in Brazil and Mexico to test the algorithm performance. We therefore created populations equivalent in size to the average dog population of states in Mexico (mean 634,361, median 409,877, simulated population 505,341), sufficient to support rabies persistence without incursions. Epidemiological parameter estimates were derived from data from Tanzania on rabid dog incubation and infection periods, and movement and biting distributions [22], that are expected to be broadly similar irrespective of the geographical setting. We modelled dog demography explicitly, tracking the number of dogs in each spatially-defined area, so that we could simulate vaccination coverage and its waning with population turnover. We heterogeneously distributed dog populations across 1km^2^ grid cells by scaling up georeferenced dog population data from Tanzania. The purpose of this was to capture reasonable population clustering known to affect rabies persistence, in the absence of detailed spatial data on dog distributions from Latin America. We assumed high dog population turnover (average lifespan of 2.5 years), which is representative of the short life-expectancy of dogs in low socio-economic settings with endemic rabies [23], and similar to those reported for populations with endemic rabies in Brazil and Mexico [24, 25]. We have previously found this model to be applicable in different settings [20, 21, 26] and we checked for appropriate dynamics (incidence patterns and persistence) prior to applying the classification algorithm.

We initialized simulations, with an average incidence of around 1% of the dog population per annum [27], with 450 cases distributed across the landscape with probability proportional to dog density. We then generated 10 stochastic realizations of 10 years of simulated cases for the following scenarios: (a) with no control measures in place, (b) with mass dog vaccination campaigns achieving an average of 60% coverage with realistic spatial heterogeneity, (c) with reduced vaccination coverage to 20% and (d) 10%. We also considered (e) incursions approximately every 6 months under high dog vaccination coverage (scenario b) and (f) reduced vaccination coverage (scenario c) and (g) into a rabies-free population. This set of 10 × 7 simulation runs each captured the full spectrum of classifications (Table 1). We then resampled the simulated time series to mimic differing levels of surveillance (i.e. 1, 2, 5, 10, 20% of circulating cases detected by the surveillance system) and applied our classification algorithm to each resampled dataset to assess the algorithm robustness to surveillance quality.

### Algorithm application

We applied our final algorithm to data from Mexico and Brazil from 2005 until 2015 in order to assess transitions from one classification to another over this period. We report these classifications (see Table 1) and their epidemiological interpretation and implications for management. We also developed a Shiny application as a dynamic interface for exploring the classification of states in Mexico and Brazil over this time period and the interpretation in terms of rabies control programme management.

## Results

### Algorithm development

On the basis of the rationale described in the methods we developed a two-stage classification process for use in settings with established dog rabies control programmes with annual or more frequent mass dog vaccination campaigns at second-level administrative units and adequate surveillance systems in place i.e. at least at Stage 3 within the SARE [28]. Our final algorithm is described in Fig. 1 and Table 2, with the temporal trend in case detection criterion applied over 5 years of monthly surveillance data.

### Algorithm testing and validation

During the algorithm development and testing we found that time series of confirmed rabies cases were highly variable (Table S1, Figures S1 and S2). Overall, we found that presence-absence time series and their trends upon logistic regression were more informative and less sensitive to biases due to population sizes or fluctuations in reporting than time series of case counts (Table S1, Figures S1 and S2 insets). Generally 4–6 years appeared to be a sufficient and useful time window to detect consistent temporal trends in case detection and be responsive to changing dynamics (Figure S3, S4 and boydorr.shinyapps.io/paho_rabies/). Using shorter time windows (< 4 years) magnified transient patterns, whereas dynamical transitions were less apparent with longer windows (> 6 years). We therefore suggest the trend in case detection criterion be applied over a 5-year time window.

Missing information is a prevalent problem in routine surveillance, potentially misleading conclusions regarding the occurrence and severity of disease circulation. We examined the effect of missing location information (major and minor administrative units corresponding to states and municipalities respectively) on classifications. Between 2005 and 2015, of 442 cases reported in Mexico 386 had no municipality information reported and 34 had no state information. Of 558 cases reported in Brazil, 326 had no municipality information reported and 14 cases had no state information. However, from 2011 onwards, municipalities and states were reported for all cases in both countries. Missing major administrative level information for cases (2.5% in Brazil, 7.6% in Mexico) did not affect the 2015 classification, but may have had a small impact in earlier years. Nonetheless the epidemiological interpretation of transitions was clear (see *Algorithm Application*).

**Fig. 2:**
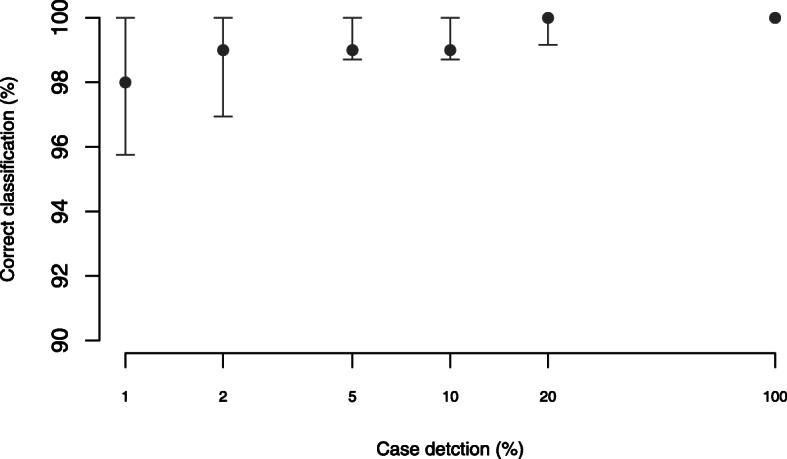
Performance of the classification algorithm according to the quality of surveillance. Note that case detection is plotted on a log scale and that the y-axis is shown from 90 to 100%. Percentage of circulating cases detected (case detection) measures surveillance quality and here relates to the extent to which suspect rabid animals are investigated thereby enabling sample collection and subsequent laboratory testing

Through subsampling simulated time series, we confirmed that our classification tool was robust in determining epidemiological status. Surveillance had to reach very low levels or be sufficiently biased for states to be misclassified (Fig. 2). In endemic settings (*Endemic/ Declining*), surveillance would need to detect less than 2.5% of all circulating cases to result in misclassification. This threshold detection level for correct classification, however, increased to 5% as incursions become the main source of cases i.e. for settings classified as *Intermittent* or *Absent-Vulnerable*.

### Algorithm application

**Fig. 3:**
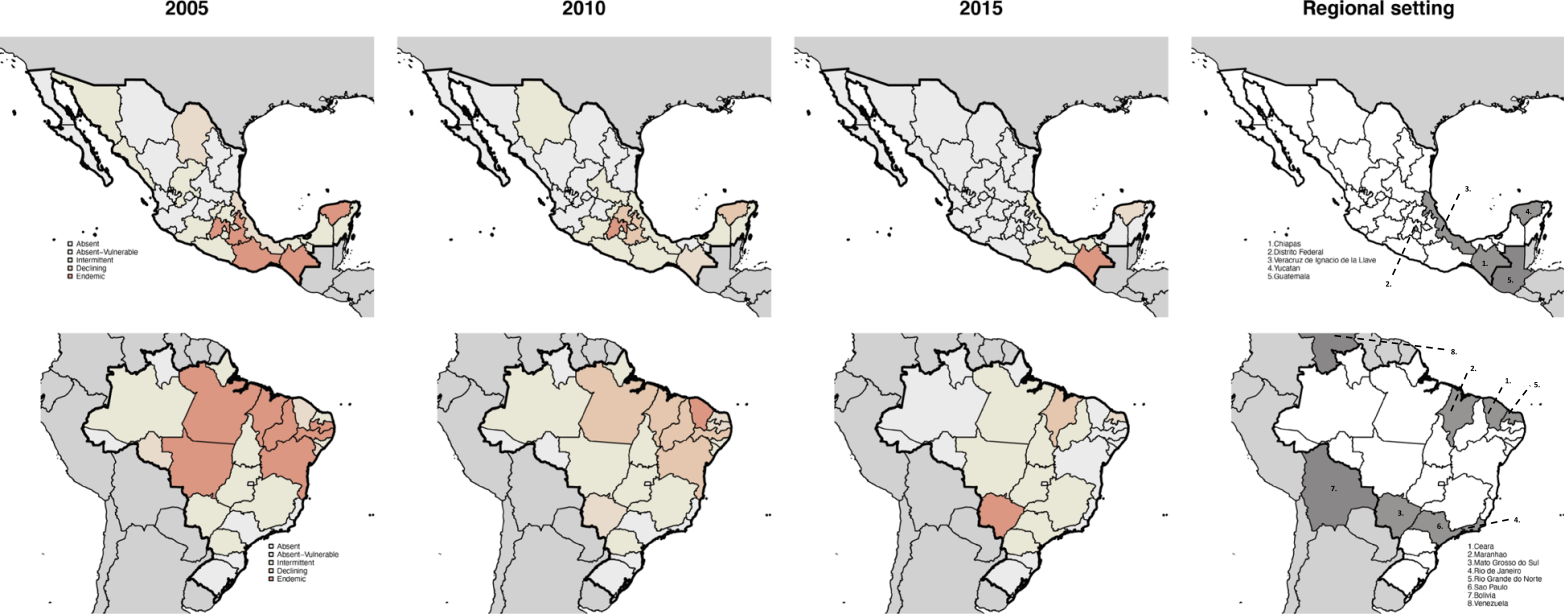
Putative classification of states in Mexico and Brazil in 2005, 2010 and 2015 (left to right). Major administrative units (states) are shaded (colour) by their epidemiological classifications. Country and state boundaries (shapefiles) were obtained from gadm.org using the *getData* function from the *raster* package in R. Grey shading shows human population density downloaded from worldpop.org and aggregated to 0.25 × 0.25 degrees per cell. The fourth panel in each row indicates epidemiologically notable states and countries frequently referred to in the main text. Note that Distrito Federal refers to Mexico City. All maps in Fig. 3 are created by the authors

Classifications of Brazilian and Mexican states in 2005, 2010 and 2015 are shown in Fig. 3 and transitions over this time period by month can be viewed via the Shiny application: https://boydorr.shinyapps.io/paho_rabies/ (and in Figures S3 and S4). Over this decade, considerable progress was evident in both countries, with many *Endemic*, *Declining* or *Intermittent* states transitioning to *Absent* or *Absent-Vulnerable.* In 2015, most states in both countries were classified as either *Absent* or *Absent-Vulnerable* (Table 3). Focal circulation persisted only in southern Mexico and northeast Brazil, except for an outbreak in Mato Grosso do Sul, Brazil in 2015 that originated from a transboundary incursion and that was rapidly controlled.

**Table 3:** Putative classification of states in Mexico and Brazil showing progression from 2005 to 2015. States presenting incursion risks highlighted in bold italics

MEXICO	2005	2010	2015
Aguascalientes	Absent	Absent	Absent
Baja California	Absent	Absent	Absent
Baja California Sur	Absent	Absent	Absent
Campeche	Absent-Vulnerable	Absent-Vulnerable	Absent
Coahuila de Zaragoza	Intermittent	Absent	Absent
Colima	Absent	Absent	Absent
***Chiapas***	***Endemic***	Intermittent	***Endemic***
Chihuahua	Absent	Absent-Vulnerable	Absent
Distrito Federal	Intermittent	Absent-Vulnerable	Absent
Durango	Absent	Absent	Absent
Guanajuato	Absent	Absent	Absent
Guerrero	Absent-Vulnerable	Absent-Vulnerable	Absent
Hidalgo	Absent-Vulnerable	***Declining***	Absent
Jalisco	Absent	Absent	Absent
Mexico	***Endemic***	***Endemic***	Absent
Michoacan de Ocampo	Absent-Vulnerable	Absent-Vulnerable	Absent
Morelos	Absent-Vulnerable	Absent-Vulnerable	Absent
Nayarit	Absent	Absent	Absent
Nuevo Leon	Absent	Absent	Absent
Oaxaca	***Endemic***	Absent-Vulnerable	Absent-Vulnerable
Puebla	***Endemic***	***Declining***	Absent
Queretaro Arteaga	Absent-Vulnerable	Absent-Vulnerable	Absent
Quintana Roo	Absent-Vulnerable	Absent-Vulnerable	Absent
San Luis Potosi	Absent	Absent-Vulnerable	Absent
Sinaloa	Absent-Vulnerable	Absent	Absent
Sonora	Absent-Vulnerable	Absent	Absent
Tabasco	Absent-Vulnerable	Absent	Absent-Vulnerable
Tamaulipas	Absent	Absent	Absent
Tlaxcala	Intermittent	Absent-Vulnerable	Absent
Veracruz de Ignacio de la Llave	Intermittent	Absent-Vulnerable	Absent-Vulnerable
Yucatan	***Endemic***	***Declining***	Intermittent
Zacatecas	Absent-Vulnerable	Absent	Absent
BRAZIL	**2005**	**2010**	**2015**
Acre	Absent	Absent	Absent
Alagoas	Absent-Vulnerable	Absent-Vulnerable	Absent
Amapa	Absent-Vulnerable	Absent-Vulnerable	Absent
Amazonas	Absent-Vulnerable	Absent-Vulnerable	Absent
Bahia	***Endemic***	***Declining***	Absent
Ceara	Intermittent	**Endemic**	Absent
Distrito Federal	Absent	Absent	Absent
Espirito Santo	Absent	Absent	Absent
Goias	Absent-Vulnerable	Absent-Vulnerable	Absent-Vulnerable
***Maranhao***	***Endemic***	***Declining***	***Declining***
Mato Grosso	***Endemic***	Absent-Vulnerable	Absent-Vulnerable
***Mato Grosso do Sul***	Absent-Vulnerable	Intermittent	***Endemic***
Minas Gerais	Absent-Vulnerable	Absent-Vulnerable	Absent-Vulnerable
Para	***Endemic***	***Declining***	Absent-Vulnerable
Paraiba	***Endemic***	Intermittent	Absent
Parana	Absent-Vulnerable	Absent-Vulnerable	Absent-Vulnerable
Pernambuco	***Endemic***	***Declining***	Absent
Piaui	***Endemic***	***Declining***	Absent-Vulnerable
Rio de Janeiro	Absent	Absent	Absent
Rio Grande do Norte	Absent-Vulnerable	Intermittent	Intermittent
Rio Grande do Sul	Absent	Absent	Absent
Rondonia	Intermittent	Absent	Absent
Roraima	Absent	Absent	Absent
Santa Catarina	Absent	Absent	Absent
Sao Paulo	Absent	Absent	Absent-Vulnerable
Sergipe	Intermittent	Absent-Vulnerable	Absent
Tocantins	Absent-Vulnerable	Absent-Vulnerable	Absent-Vulnerable

In Mexico, only Chiapas state on the border with Guatemala was classified as *Endemic* in 2015 (Fig. 3). Yucatán state was classified as *Intermittent* rather than *Declining*, because one of the two detected cases in the prior 2 years (2014) was a wildlife variant. Incursion risks generally declined as rabies was controlled in central Mexico, primarily in Mexico City and surrounding states of Puebla and Veracruz (Fig. 3), but circulation in Chiapas and neighbouring Guatemala still put other states at risk of re-emergence (Fig. 3a, b). Yucatán is one of the few states that switched from a putatively *Absent* classification back to *Endemic* indicating that investigation, possibly into wildlife circulation is warranted (Figure S3). A prolonged absence in Chiapas led to its reclassification from *Endemic* to *Absent-Vulnerable* in 2006–2007 prior to detection of rabies again in 2008 and reversion to *Endemic* (Figure S3). It is unclear whether these transitions in Chiapas were the result of improved surveillance or incursions from Guatemala. Missing data on locations of cases during this period may have affected the earlier classifications.

In 2015 in Brazil, only Mato Grosso do Sul was classified as *Endemic* as a consequence of the outbreak in late 2015, while in Northeast Brazil, Maranhão and Rio Grande do Norte were classified as *Declining* and *Intermittent* respectively*.* Incursion risks are largely driven by rabies circulation in Northeastern states where until recently dog rabies variants co-circulated together with wildlife variants (predominantly in Rio Grande de Norte, Ceará and Sergipe); however, continued dog rabies control efforts appear to have reduced circulation to just Maranhão and possibly Rio Grande de Norte. The outbreak in Mato Grosso do Sul was detected in Corumba, a border town with Bolivia, and was restricted to the municipalities of Corumba and Ladario (Fig. 3). São Paulo State was initially classified as *Intermittent*, but variant information indicated that recent cases were associated with wildlife variants rather than canine rabies (V1 or V2).

**Fig. 4:**
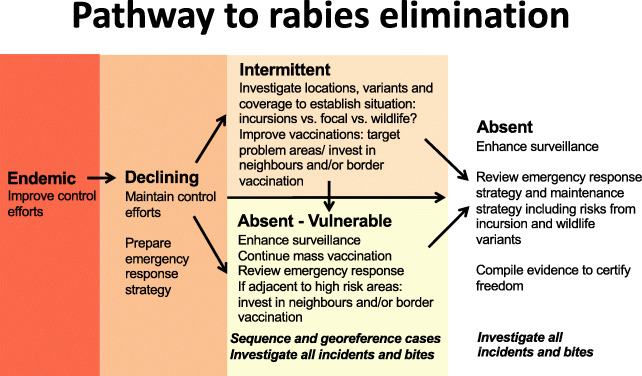
Putative epidemiological classifications and associated management actions for progression towards elimination

We derived management guidance for each classification, summarized in Fig. 4. The Shiny app that we developed also visualizes progress towards elimination across the region, and was designed to communicate tailored guidance on rabies management at the state level: https://boydorr.shinyapps.io/paho_rabies/.

## Discussion

### Key findings

Guidance for established rabies elimination programmes, such as those in Latin America, is relatively limited and not geographically specific [10, 29]. The management tool that we have developed and validated using surveillance data from Mexico and Brazil allows to identify mutually exclusive epidemiological situations from a simple algorithm, without the need for extensive statistical expertise (illustrated at: https://boydorr.shinyapps.io/paho_rabies/). By classifying states in these countries, we determined surveillance and control priorities at local, national and regional levels, and derived tailored guidance on how to continue progressing towards elimination, while revealing insights into rabies dynamics (Figs. 3 and 4, Table 4).

**Table 4:** Management guidance based on classifications

1) **ENDEMIC TRANSMISSION:** control measures have not been implemented sufficiently to demonstrably reduce incidence. Mass vaccination campaigns should therefore be implemented within all administrative units, at least annually, using modern cell-culture vaccines of proven efficacy, aiming to reach > 70% of dogs in all communities. Gaps in coverage can allow persistence, even when average coverage is high [21, 26]. Therefore, if ongoing vaccination campaigns are not controlling rabies, their implementation and coverage at local levels should be monitored to identify areas for improvement. 2) **DECLINING TRANSMISSION:** Control measures are demonstrably effective and current mass dog vaccinations should be sustained. Plans for maintaining rabies freedom should be developed, including emergency response strategies and preparation for enhanced surveillance required to verify freedom from disease [27]. 3) **INTERMITTENT DETECTION**: Criteria indicate that either: i) transmission is endemic but surveillance is poor; ii) transmission has been interrupted but incursions are frequent; or iii) other circulating variants are causing cases in dogs. Assuming surveillance information is available, updating the classification with removal of wildlife variants could resolve scenario *iii*, while case locations may allow incursions to be distinguished from local transmission (i.e. scenario *ii*); otherwise surveillance needs enhancing to distinguish these scenarios. Management recommendations are either for improved high coverage comprehensive vaccination campaigns to interrupt transmission (scenario *i*); investment in rabies control in source populations and in populations at risk from incursions (scenario *ii*); maintained dog vaccination to prevent further spread of these spillover variants (scenario *iii*). 4) **ABSENT-VULNERABLE:** Control efforts should be maintained while incursion risks remain high. Enhanced surveillance should be implemented for early detection of incursions and a detailed emergency response strategy prepared to ensure rapid response capacity [20]. In light of any incursions this emergency response strategy should be reviewed. All cases should be sequenced to identify variants and sources of incursions. Evidence should be compiled to verify freedom from rabies, including the absence of case detection during 2 years of enhanced surveillance [20]. 5) **ABSENT:** Although no cases have been detected for extended periods, enhanced surveillance should be maintained and evidence compiled to verify rabies freedom.	

Progress towards elimination was evident in both Mexico and Brazil; however, focal transmission remains a threat for re-emergence in ostensibly rabies-free states. Connectivity has been demonstrated to play a critical role in rabies persistence, with infection maintained across, and driven by, large interconnected metapopulations (rather than dense conurbations) [30, 31]. Our classification identified metapopulations that support focal transmission, such as in central Mexico in the recent past (e.g. in 2010, Fig. 3), and the cross-border area between Mexico and Guatemala, as well as in Northeast Brazil (Fig. 3). There is an urgent need to improve surveillance and control in remaining foci, including endemic bordering countries. For example, the outbreak in the municipality of Corumba, Mato Grosso do Sol, on the Brazil-Bolivia border is rumoured to have spread from Bolivia, even though no cases were reported to SIRVERA from Bolivia in over 3 years. Incursions with long-lasting consequences have been reported elsewhere in Latin America [32], and globally [20, 33], and threaten the regional elimination effort. Moreover, rabies still persists in a few states where control programmes have been ongoing for decades. We suggest that in these *Endemic* states, post-vaccination monitoring is needed to identify the causes of slow progress, which likely relate to inadequacies in dog vaccination campaigns. Vaccination coverage in Latin America has generally been estimated retrospectively from human:dog ratios. However, human:dog ratios can be heterogeneous and change considerably over time [34]. Estimating numbers of distinct dogs vaccinated can also be difficult, as some dogs are vaccinated repeatedly during outbreak responses unless concerted efforts are made to target areas missed during campaigns. Gaps in coverage were previously found to be a critical factor in rabies control, prolonging progress towards elimination [20, 26]. Post-campaign assessments of coverage are needed to both identify and remedy problematic areas [35]. Strengthening the delivery and monitoring of dog vaccination campaigns in areas with focal transmission (including in neighbouring countries), is likely to be the single most important programmatic change for improving elimination prospects regionally.

### Strengths and limitations

Surveillance quality affects the utility of evaluation tools to inform management. We considered surveillance quality to be sufficient throughout Mexico and Brazil based on assessment of indicators from the last 5 years including annual dog vaccinations in every state, adequate sample submissions and regular proficiency testing of laboratories [36], further supported by interviews with rabies programme managers and through PAHO missions. For other countries with less progressed control programmes and weaker surveillance, application of this tool would not be appropriate. For example, settings where locally-acquired human rabies cases are reported in the absence of confirmed animal cases would be indicative of inadequate surveillance. A particular concern is that the absence of detected cases reflects weak surveillance and not elimination. Although our requirement for a decline in case detection concomitant with dog vaccination prior to classification to putatively *Absent* guards against this, we recommend initial review to determine whether a country would benefit from this tool, for example, progression on SARE to at least step 3. Most countries in sub-Saharan Africa and Asia are probably at too early a stage in their control efforts for this tool to yet be of use, but we anticipate that in Latin America this tool could be applied to most countries and its use would complement SIRVERA.

Using sub-sampled simulated data, we demonstrated the extent to which our tool was robust to surveillance quality in endemic settings (Fig. 2). Surveillance had to reach very low levels or be very biased for states to be misclassified (< 2.5% detection in *Endemic/Declining* classifications). This threshold detection level increased to 5% as incursions become the main source of cases i.e. in *Intermittent* and *Absent-Vulnerable* classifications. We therefore emphasize the need to enhance surveillance to detect at least 5% of circulating cases, a recommendation consistent with prior work showing that 5% case detection is required to verify disease freedom [9]. Integrated Bite Case Management shows promise as a tool that can detect > 10% of circulating cases [27], but further work is needed to assess its feasibility for implementation across a wide range of settings. We also emphasize that classification of *Absent-Vulnerable* and *Absent* using this tool is only putative and does not certify or guarantee rabies-free status. However, we consider that this classification would be a strong indication of readiness, when a country or state is well positioned to compile evidence of freedom in line with OIE requirements [11].

We recommend applying this tool over a five-year window of surveillance data, as shorter periods tend to magnify transient patterns and longer windows are potentially less responsive to epidemiological transitions. Case data should be reported by state and municipality, as this also provides a simple criterion for assessing surveillance quality. In Mexico and Brazil, case locations were complete at state-level from 2011 onwards and therefore did not affect later classifications; however, classifications earlier in the study may have been affected by this missing information. Municipality information could also be useful as elimination is approached; in fact, we suggest that incorporating municipality information into inference approaches could generate a better understanding of incursion risks and more tailored management recommendations to prevent them. Future work testing the application of this tool in other countries and at other spatial scales would be valuable to better understand its utility.

### Surveillance implications

With the progressive control and elimination of dog-mediated rabies from the region, other circulating virus variants have become increasingly apparent [37–39]. Variant identification played a discriminatory role for our classifications of states in both Mexico and Brazil, where both dog- and wildlife-associated variants have co-occurred. Circulation of wildlife variants is not necessarily an obstacle to elimination of dog rabies variants, but wildlife variants have potential to spread in the dog population and pose public health risks. Strategies for maintaining rabies freedom and for judicious use of post-exposure prophylaxis therefore need to account for these potentially complex situations, which may affect the scaling back of dog vaccination and protocols for identifying and treating bite victims. We therefore recommend that as states approach elimination (*Intermittent, Absent-Vulnerable* or *Absent* classification), sequencing of all detected cases be undertaken (Fig. 4). Since only very few cases are detected in these situations, this should not be cost prohibitive, though, baseline characterization of historically circulating variants is required.

The target for both Mexico and Brazil is now nationwide interruption of transmission and verification of disease freedom. For diseases that have been eradicated or regionally eliminated, intensified surveillance approaches have been employed to increase case detection [40–42]. Such approaches are now urgently required for areas classified as *Absent* or *Absent-Vulnerable*, or with *Intermittent* detection, to resolve uncertainties regarding viral circulation, initiate early outbreak responses and verify freedom (Table 4). We suggest that surveillance guided by Integrated Bite Case Management [43], with epidemiological investigations triggered by bites from suspicious animals, should enable verification of rabies freedom and guide scaling back of mass dog vaccination [27]. This approach should also help to identify transmission of other rabies variants and ensure appropriate treatment for exposed persons [44]. Moreover, this approach should result in sample submissions, as well as observation or quarantining of biting animals [45] which are important surveillance indicators. Likewise, genomic surveillance is a valuable tool [46], that used in combination with epidemiological data, could be crucial in guiding the rabies endgame. Sequencing of viruses can resolve key questions about viral circulation, discriminating wildlife variants from dog variants [37], including the potential for host shifts [47], and providing insights about the persistent lineages in remaining foci [48], which would be useful now in Chiapas state, Mexico and in Guatemala. In a previously rabies-free area, sequencing could identify the source(s) of incursions [49], and confirm that the new virus lineage differed from those historically circulating (i.e. undetected endemic circulation) [30, 50]. Adding functionality within SIRVERA for mapping georeferenced sequence data could facilitate rapid assessment of circulating viral lineages and potential incursion threats. Finally, contingency plans are urgently needed for states approaching elimination, and these should be regularly reviewed to ensure response capacity is maintained (Fig. 4).

## Conclusion

The management tool we have developed adds to an increasing toolbox available to rabies managers to support them in determining their progress towards elimination, while providing tailored guidance for different epidemiological situations based on objective criteria derived from routine surveillance data. The added value of this tool is the direct use of surveillance data to provide quantitative measures of progress, but as a consequence this tool can only be applied to states with established surveillance capacity. The resulting management recommendations from applying this tool are logical and may appear self-evident (Fig. 4). But in practice, budget and human resource for post-vaccination monitoring and surveillance activities are limited, and often are not undertaken unless a strong case can be made as to their importance. This tool provides this critical evidence in a geographically targeted way. In summary, we identified the key remaining challenges to elimination of dog-mediated rabies from Mexico and Brazil as: 1) interruption of transmission from focally persistent states/borders that pose wider incursion risks; and 2) enhancing surveillance to distinguish variants, respond to and minimize incursion risks and verify freedom from disease, in order to allow relaxation of control measures without risking re-emergence. Application of this management tool in Latin America could be used to prioritize efforts to accelerate progress towards regional elimination and ensure readiness for verification and maintenance of rabies freedom. Incorporation of this tool into the SIRVERA platform would encourage further engagement and use by programme managers. More broadly, we suggest this tool to be adapted and used effectively in other parts of the world to guide progress towards the global elimination of dog-mediated rabies.

## Supplementary information

**Additional file 1.**

**Additional file 2.**

**Additional file 3.**

**Additional file 4.**

**Additional file 5.**

## Data Availability

The datasets (figures and code) generated and/or analysed during the current study are available in a private GitHub repository, and available from the corresponding author on reasonable request.

## Abbreviations

PAHO: the Pan American Health Organization
SIRVERA: the Regional Epidemiologic Surveillance System for Rabies
SARE: Stepwise Approach towards Rabies Elimination
