# Supplementary figures and images for: Towards the elimination of dog-mediated rabies: development and application of an evidence-based management tool
Source: BMC Infect Dis. 2020 Oct 20;20:778. doi: 10.1186/s12879-020-05457-x (PMC7574347; doi:10.1186/s12879-020-05457-x)

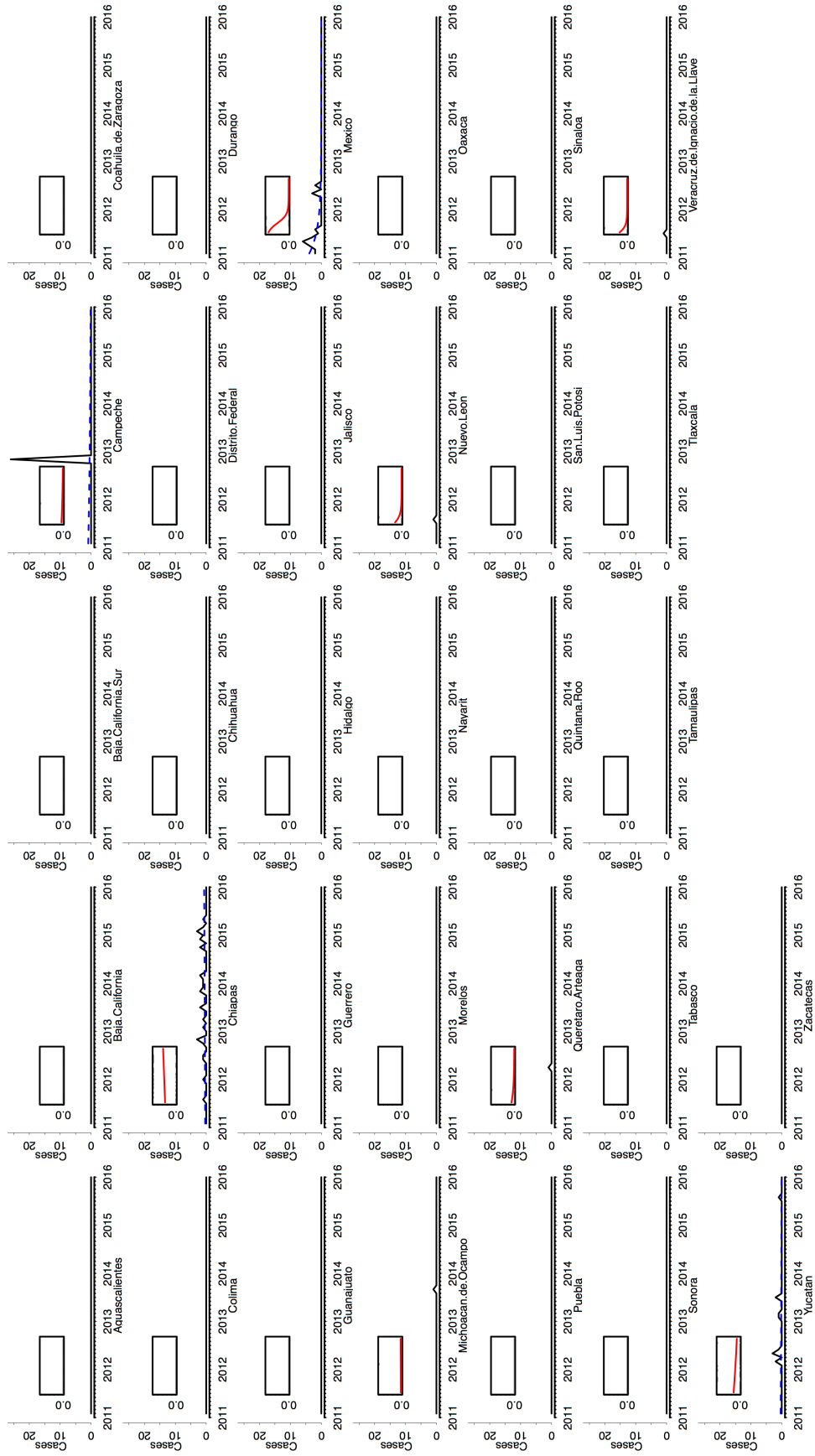

Supplement: Supplementary file 1 — Additional file 1. [file 12879_2020_5457_MOESM1_ESM.pdf]

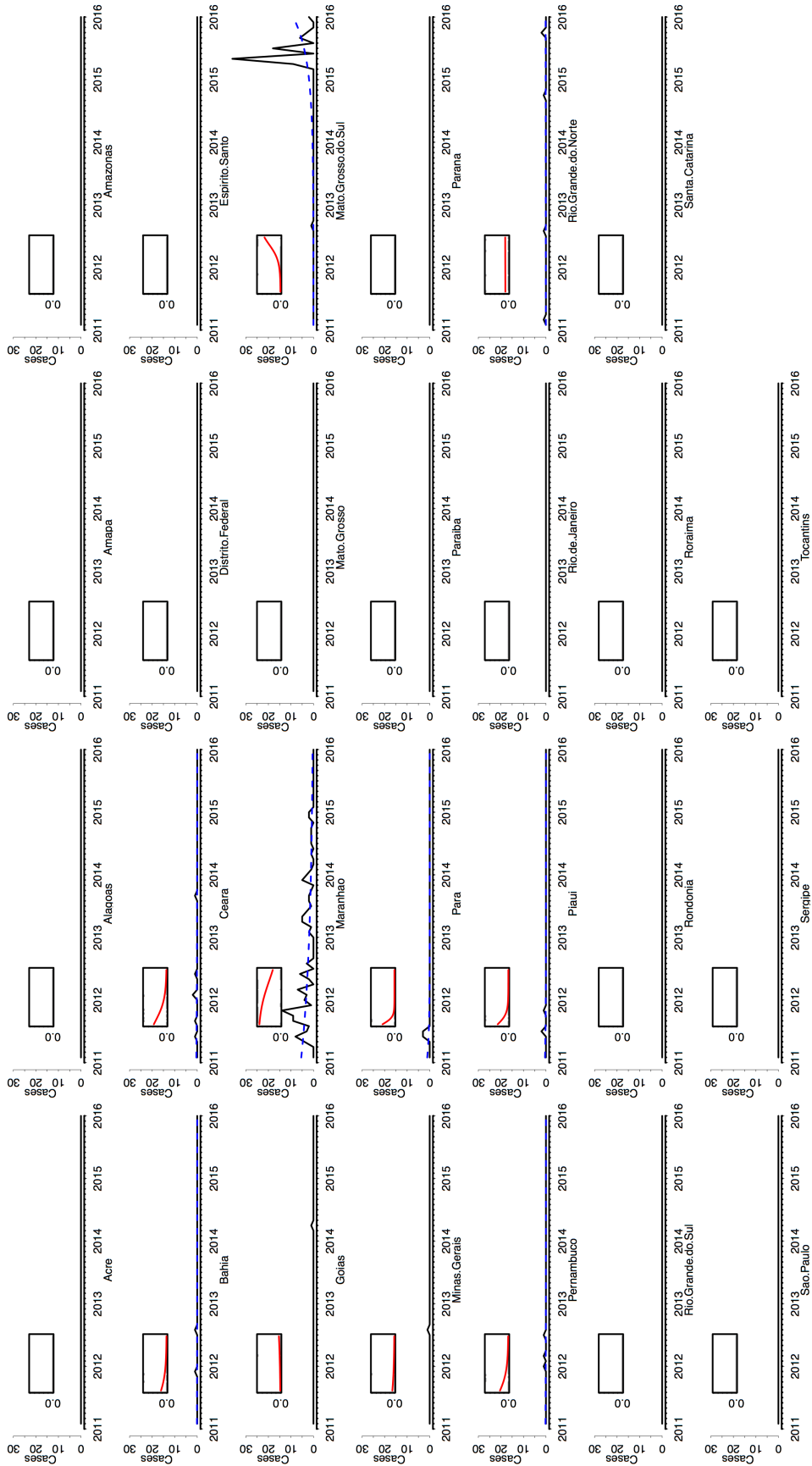

Supplement: Supplementary file 2 — Additional file 2. [file 12879_2020_5457_MOESM2_ESM.pdf]

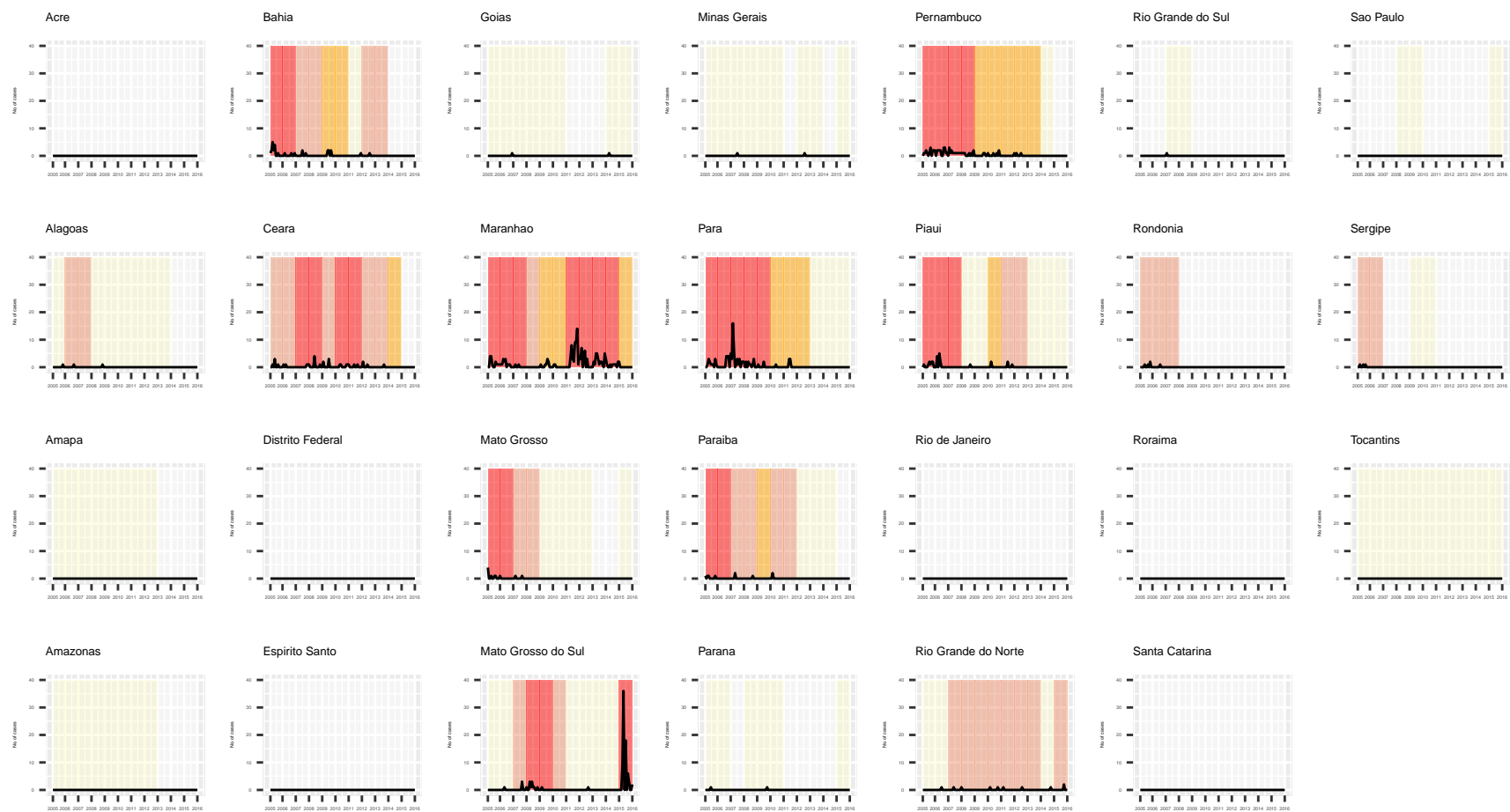

Supplement: Supplementary file 4 — Additional file 4. [file 12879_2020_5457_MOESM4_ESM.pdf]
