# Supplementary material for: Towards the elimination of dog-mediated rabies: development and application of an evidence-based management tool
Source: BMC Infect Dis. 2020 Oct 20;20:778. doi: 10.1186/s12879-020-05457-x (PMC7574347; doi:10.1186/s12879-020-05457-x)
Supplement: Supplementary file 3 — Additional file 3. [file 12879_2020_5457_MOESM3_ESM.pdf]

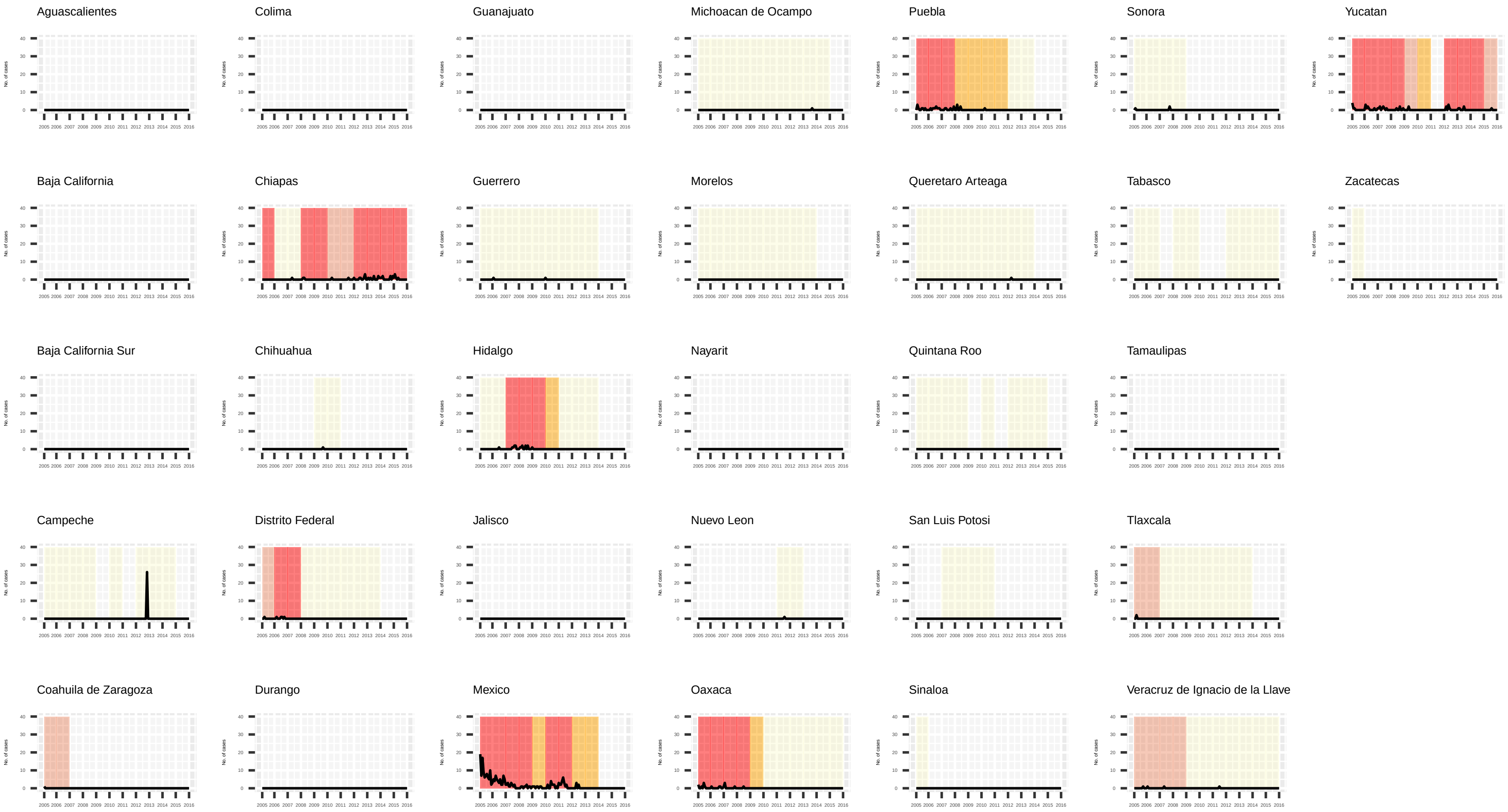

Aguascalientes
Colima
Guanajuato
Michoacan de Ocampo
Puebla
Sonora
Yucatan
Baja California
Chiapas
Guerrero
Morelos
Queretaro Arteaga
Tabasco
Zacatecas
Baja California Sur
Chihuahua
Hidalgo
Nayarit
Quintana Roo
Tamaulipas
Campeche
Distrito Federal
Jalisco
Nuevo Leon
San Luis Potosi
Tlaxcala
Coahuila de Zaragoza
Durango
Mexico
Oaxaca
Sinaloa
Veracruz de Ignacio de la Llave
No. of cases
0 10 20 30 40
2005 2006 2007 2008 2009 2010 2011 2012 2013 2014 2015 2016
